# The Influence of Specific Pathogen-Free and Conventional Environments on the Hematological Parameters of Pigs Bred for Xenotransplantation

**DOI:** 10.3390/life14091132

**Published:** 2024-09-08

**Authors:** Won Kil Lee, Hwi-Cheul Lee, Seunghoon Lee, Haesun Lee, Sang Eun Kim, Minguk Lee, Jin-Gu No, Keon Bong Oh, Poongyeon Lee

**Affiliations:** Animal Biotechnology Division, National Institute of Animal Science, Rural Development Administration, Wanju-gun 55365, Jeollabuk-do, Republic of Korea; lwk2737@korea.kr (W.K.L.); hclee@korea.kr (H.-C.L.); sage@korea.kr (S.L.); leehs1498@korea.kr (H.L.); sesesese@korea.kr (S.E.K.); bosuk7842@korea.kr (M.L.); shrkftm@korea.kr (J.-G.N.)

**Keywords:** specific pathogen-free pig, hematological parameters, xenotransplantation

## Abstract

Blood analysis plays a pivotal role in assessing the health of laboratory animals, including pigs. This study investigated the hematological profiles of transgenic pigs of the MGH breed for xenotransplantation, focusing on the effect of housing conditions on blood parameters. A cohort of pigs was longitudinally monitored from 6 to 18 months of age in both conventional and specific pathogen-free (SPF) environments. Red blood cells (RBCs), hemoglobin (HGB), and white blood cells (WBCs) were analyzed using standardized hematology analyzers. The results revealed that RBC and HGB levels were consistently higher in SPF-housed pigs. Notably, WBC counts were significantly lower in SPF-housed pigs, suggesting that reduced pathogen exposure under SPF conditions effectively diminished immune system activation. These findings raise a novel question as to whether distinct hematological parameters of specific and/or designated PF pigs would be advantages for the success of clinical xenotransplantation trials.

## 1. Introduction

Blood analysis plays a pivotal role in veterinary and medical sciences, especially in assessing the health of laboratory animals, such as pigs. These animals are central to transplantation research and drug development due to their physiological similarities to humans. Analyzing key components such as red blood cells (RBCs), hemoglobin (HGB), and white blood cells (WBCs) offers critical insights into the physiological state of these animals. Variability in blood cell counts may be influenced by factors such as breed, age, environment, and overall health status, underscoring the importance of establishing specific hematological baselines for different conditions [1,2].

Pig xenografts are becoming increasingly prevalent in the current clinical trial era [3,4], and the use of designated pathogen-free (DPF) pigs is essential for such applications [5]. We examined the hematological profiles of pigs housed in specific pathogen-free (SPF) and conventional conditions, which were specifically bred for xenotransplantation research [6]. The results revealed distinct hematological parameters in SPF pigs, raising a question as to how differences in physiology between pigs housed in filtered air with positive pressure and in natural conditions impact clinical trials in xenotransplantation.

## 2. Materials and Methods

### 2.1. Ethics Statement

The ethical approval for this study was granted by the Animal Experimental Ethics Committee of the National Institute of Animal Science, and all procedures were conducted in compliance with established ethical standards (approval number: NIAS 2020-0415). 

### 2.2. Animals

Alpha-1,3-galactosyltransferase-deficient and human membrane cofactor protein-expressing (GTKO/MCP) transgenic (TG) pigs of the MGH breed [7] were initially selected at the age of 6 months from two distinct housing environments, 58 conventional (37 males and 21 females) and 13 SPF (6 males and 7 females) pigs. Each pig was rigorously screened for health conditions and pregnancy, with any individuals manifesting signs of illness or pregnancy being promptly excluded from the study. These selected pigs were then continuously housed under their respective conditions throughout the study period. The experimental design was a longitudinal study in which the same group of pigs was monitored over time to track hematological changes at the initial age of 6 months and subsequently at 12 and 18 months. The SPF facility was maintained with high-efficiency particulate air filters at positive pressure, and the pigs were provided with an irradiated pig pellet diet. The pigs housed in SPF were free of 42 pathogens (Table S1). No new animals were introduced to the cohort after the initial selection at 6 months. 

### 2.3. Complete Blood Cell (CBC) Analysis 

Blood samples for CBC analysis were collected from the jugular vein using an 18-gauge needle. The blood was immediately placed into a BD Vacutainer^®^ containing ethylene diamine tetra acetic acid to prevent clotting (BD, Franklin Lakes, NJ, USA). The samples were promptly transported to the laboratory for further analysis. RBC, HGB, and WBC counts were determined using a Procyte DMTM analyzer (IDEXX, Westbrook, ME, USA). The top and bottom 5% of the distribution curves were removed for each measured parameter to analyze hematological data from pigs housed in conventional facilities.

### 2.4. Statistical Analysis

The levels of RBCs, HGB, and WBCs were analyzed using GraphPad Prism 5 (GraphPad Software, La Jolla, CA, USA). All the data are presented as means ± standard deviation and were analyzed using Student’s *t*-test to test the significance of pig blood (*p* < 0.05).

## 3. Results and Discussion

The first longitudinal analysis of comprehensive hematological parameters in the miniature pig breed, MGH, was conducted on TG pigs housed under both conventional and SPF conditions. Conventional TG pigs typically maintain RBC and HGB levels close to the upper limit of the normal range. By contrast, WBC counts were consistently higher in TG pigs housed in conventional facilities across all developmental stages, significantly exceeding the normal range (Table 1). Previous studies with a genetically identical strain of miniature pigs demonstrated significantly higher RBC and WBC counts than full-sized farm breeds under conventional conditions [8]. Similarly, other miniature pig breeds, such as Yucatan [9], Bama Xiang [10], and Microminipig [11], also exhibit hematological levels above normal ranges, indicating a consistent trend across different miniature breeds, whether transgenic or wild type. A hybrid breed developed by combining a miniature of GTKO background with a wild-type farm breed demonstrated hematological patterns of that farm breeds (unpublished data). 

**Figure 1:**
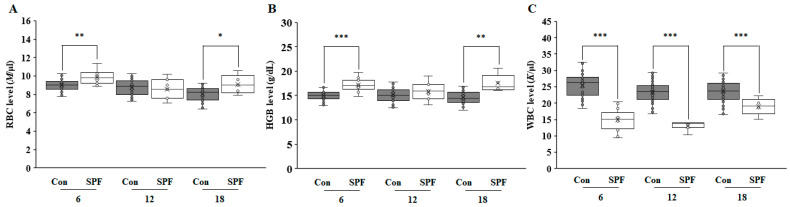
Hematological profiles of GTKO/MCP transgenic pigs over time in different housing environments. Panels show levels of red blood cells (RBC, Panel (**A**)), hemoglobin (HGB, Panel (**B**)), and white blood cells (WBC, Panel (**C**)) at 6, 12, and 18 months in conventional (Con) and specific pathogen-free (SPF) facilities. The values are presented as mean ± SD, with sample sizes at each time point indicated as follows: at 6 months (Con: n = 58, SPF: n = 13), at 12 months (Con: n = 52, SPF: n = 7), and at 18 months (Con: 49, SPF: n = 5). These numbers reflect the volume of pigs analyzed in each housing condition at every specified age point. Asterisks indicate statistical significance compared to the conventional facility at the same time point (* *p* < 0.05, ** *p* < 0.01, *** *p* < 0.001).

Figure 1 illustrates the significant differences between the two types of facilities. Pigs in SPF facilities displayed higher RBC and HGB levels and lower WBC counts across all age groups than those in conventional facilities. This pattern mirrors findings in full-sized farm pig breeds, such as the Large White [12], suggesting that SPF environments influence hematological parameters irrespective of the pig breed. WBC counts are crucial for immune responses and reflect changes in health status due to age or environmental conditions [13,14]. Supplementary Figure S1 supports this observation, showing a notable decrease in lymphocyte counts in SPF-housed pigs at 6, 12, and 18 months. This reduction in lymphocytes and WBCs likely reflects the diminished immunogenic stress associated with reduced pathogen exposure in SPF environments, as suggested by recent studies on gut microbiota diversity (accepted in J Microbiol and Biotech). Hemoglobin, a key component of RBCs, is well known to be essential for the transport of oxygen and carbon dioxide and is positively associated with blood pressure in healthy individuals [15]. The reasons for higher RBC and HGB levels in SPF-housed TG pigs and their effects on health remain unclear, especially because no abnormalities in growth, physical health, or reproduction were observed in these pigs.

Due to space limitations in the SPF facility, this current study was performed with a restricted number of pigs, including the top and bottom 5% of the levels. The levels in 12-month-old pigs were lower than those in six and 18 months. A larger number of pigs may demonstrate more consistent hematological patterns. Nevertheless, the findings suggest that pigs in SPF and DPF facilities generally exhibit higher RBC and HGB levels and lower WBC counts than pigs under natural conditions. Whether these specific hematological changes offer advantages or disadvantages for the success of clinical xenotransplantation trials remains to be determined.

## 4. Conclusions

This study’s findings indicate that SPF conditions significantly alter the hematological profiles of transgenic pigs, with increased levels of RBCs and HGB and decreased WBC counts compared to those housed in conventional environments. These hematological differences underscore the potential advantages of SPF conditions in reducing immune activation, which is crucial for the success of xenotransplantation, as it could lead to lower rates of infection and graft rejection. However, the impact of these physiological changes on the overall success of xenotransplantation remains uncertain. This necessitates further investigation to determine whether the altered immune environment in SPF pigs translates into improved clinical outcomes in xenotransplantation trials. This study highlights the complexity of transplant biology and underscores the need for a thorough understanding of how environmental factors influence the health and suitability of transgenic pigs for clinical applications. The results contribute significantly to the field of transplantation medicine, offering insights that could help optimize the preparation of animal models for clinical use, thereby enhancing the success rates of xenotransplantation procedures and improving patient outcomes.

## Figures and Tables

**Table 1:** Hematological profiles of transgenic pigs of the MGH breed in conventional facilities.

Facilities	Month	No. of Pig	RBC (M/uL)			HGB (g/dL)			WBC (k/uL)		
			Mean ± SD	Min	Max	Mean ± SD	Min	Max	Mean ± SD	Min	Max
Convent-ional	6	58	8.99 ± 0.59	7.78	10.26	14.92 ± 0.91	12.90	16.70	25.44 ± 3.51	18.37	32.45
	12	52	8.76 ± 0.82	7.29	10.28	15.15 ± 1.39	12.50	17.80	23.33 ± 3.08	16.82	29.45
	18	49	8.03 ± 0.73	6.43	9.24	14.53 ± 1.27	12.00	16.90	23.72 ± 3.34	16.56	29.14
Normal reference *				5.00	8.00		10.70	16.70		11.00	22.00

* provided by analyzer.

## Data Availability

Data are contained within the article or Supplementary Table and Figure.

## Supplementary Materials

The following supporting information can be downloaded at https://www.mdpi.com/article/10.3390/life14091132/s1, Table S1: List of pathogens excluded from SPF pigs, Figure S1: Comparative Analysis of Lymphocyte Counts in GTKO/MCP Transgenic Pigs Over Time in Different Housing Environments.

## References

[1] Burks M.F., Tumbleson M.E., Hicklin K.W., Hutcheson D.P., Middleton C.C. (1977). Age and sex related changes of hematologic parameters in Sinclair(S-1) miniature swine. Growth.

[2] Fisher D.D., Wilson L.L., Scholz R.W. (1980). Environmental and genetic effects on hematologic characteristics of beef cows. Am. J. Vet. Res..

[3] Montgomery R.A., Stern J.M., Lonze B.E., Tatapudi V.S., Mangiola M., Wu M., Weldon E., Lawson N., Deterville C., Dieter R.A. (2022). Results of Two Cases of Pig-to-Human Kidney Xenotransplantation. N. Engl. J. Med..

[4] Moazami N., Stern J.M., Khalil K., Kim J.I., Narula N., Mangiola M., Weldon E.P., Kagermazova L., James L., Lawson N. (2023). Pig-to-human heart xenotransplantation in two recently deceased human recipients. Nat. Med..

[5] Hering B.J., Cozzi E., Spizzo T., Cowan P.J., Rayat G.R., Cooper D.K., Denner J. (2016). First update of the International Xenotransplantation Association consensus statement on conditions for undertaking clinical trials of porcine islet products in type 1 diabetes—Executive summary. Xenotransplantation.

[6] Hwang S., Oh K.B., Kwon D.-J., Ock S.-A., Lee J.-W., Im G.-S., Lee S.-S., Lee K., Park J.-K. (2013). Improvement of cloning efficiency in minipigs using post-thawed donor cells treated with roscovitine. Mol. Biotechnol..

[7] Sachs D.H., Leight G., Cone J., Schwarz S., Stuart L., Rosenberg S. (1976). Transplantation in miniature swine. I. Fixation of the major histocompatibility complex. Transplantation.

[8] Cho A., Oh K.B., Roh J.-H., Jung Y.-H., Jung S.-H., Kang M.-G., Kim M.-S., Do Y.J., Oh S.-I., Kim E. (2019). Comparison of hematological values of conventional pigs and transgenic pigs supressed in immune rejection response. Korean J. Vet. Serv..

[9] Rispat G., Slaoui M., Weber D., Salemink P., Berthoux C., Shrivastava R. (1993). Haematological and plasma biochemical values for healthy Yucatan micropigs. Lab. Anim..

[10] Mo J., Lu Y., Xing T., Xu D., Zhang K., Zhang S., Wang Y., Yan G., Lan G., Liang J. (2022). Blood metabolic and physiological profiles of Bama miniature pigs at different growth stages. Porc. Health Manag..

[11] Kawaguchi H., Yamada T., Miura N., Takahashi Y., Yoshikawa T., Izumi H., Kawarasaki T., Miyoshi N., Tanimoto A. (2012). Reference values of hematological and biochemical parameters for the world smallest microminipigs. J. Vet. Med. Sci..

[12] Radulovic E., Mehinagic K., Wüthrich T., Hilty M., Posthaus H., Summerfield A., Ruggli N., Benarafa C. (2022). The baseline immunological and hygienic status of pigs impact disease severity of African swine fever. PLoS Pathog..

[13] Friendship R.M., Lumsden J.H., McMillan I., Wilson M.R. (1984). Hematology and biochemistry reference values for Ontario swine. Can. J. Comp. Med..

[14] Faustini M., Munari E., Colombani C., Russo V., Maffeo G., Vigo D. (2000). Haematology and plasma biochemistry of Stamboek pre-pubertal gilts in Italy: Reference values. J. Vet. Med. A.

[15] Atsma F., Veldhuizen I., de Kort W., van Kraaij M., Jong P.P.-D., Deinum J. (2012). Hemoglobin level is positively associated with blood pressure in a large cohort of healthy individuals. Hypertension.

