# Supplementary material for: The Influence of Specific Pathogen-Free and Conventional Environments on the Hematological Parameters of Pigs Bred for Xenotransplantation
Source: Life (Basel). 2024 Sep 8;14(9):1132. doi: 10.3390/life14091132 (PMC11433355; doi:10.3390/life14091132)
Supplement: Supplementary file 1 [file life-14-01132-s001.zip › life-3101994-supplementary Figure.pptx]

## Slide 1
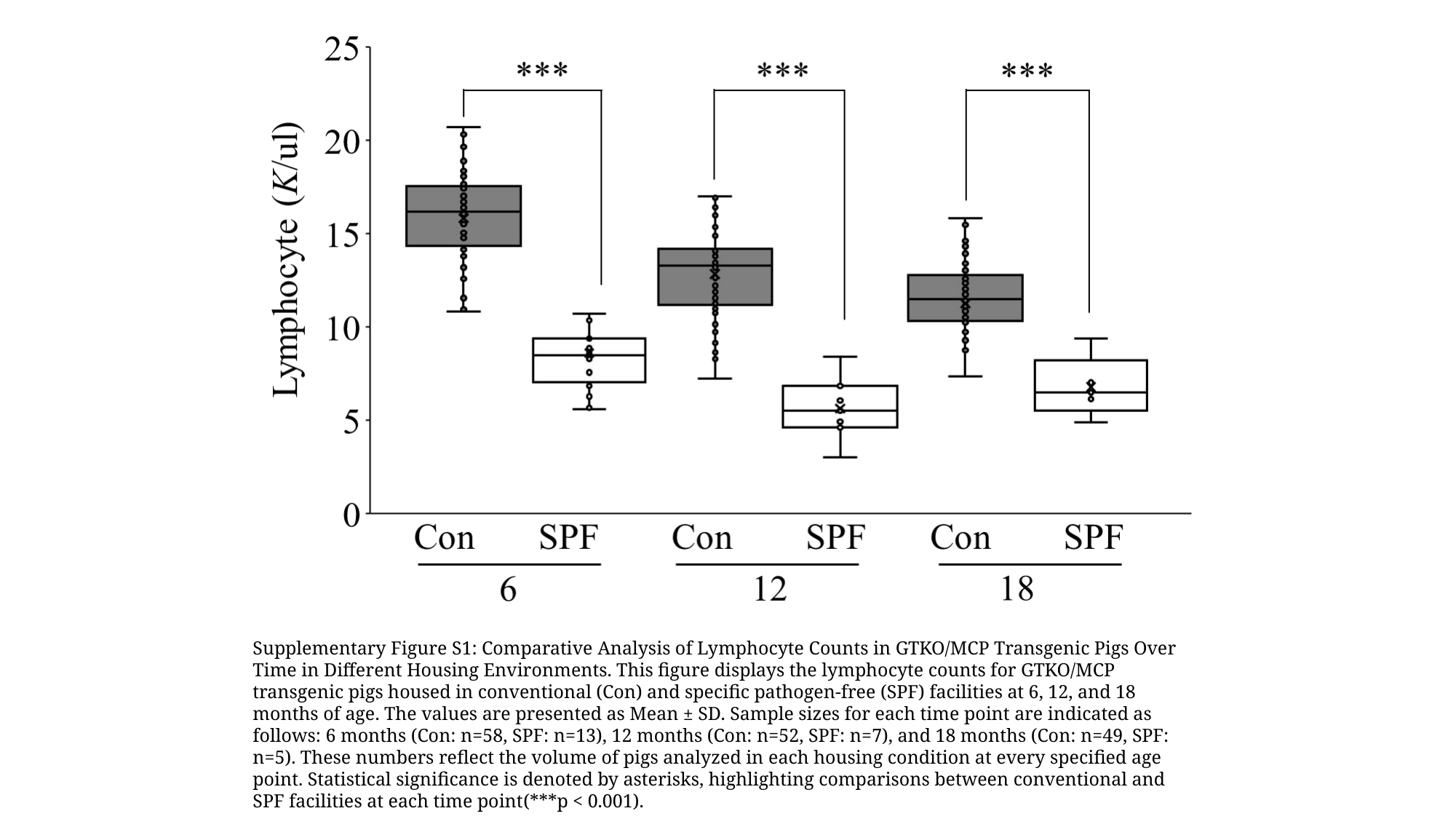

Supplementary Figure S1: Comparative Analysis of Lymphocyte Counts in GTKO/MCP Transgenic Pigs Over Time in Different Housing Environments. This figure displays the lymphocyte counts for GTKO/MCP transgenic pigs housed in conventional (Con) and specific pathogen-free (SPF) facilities at 6, 12, and 18 months of age. The values are presented as Mean ± SD. Sample sizes for each time point are indicated as follows: 6 months (Con: n=58, SPF: n=13), 12 months (Con: n=52, SPF: n=7), and 18 months (Con: n=49, SPF: n=5). These numbers reflect the volume of pigs analyzed in each housing condition at every specified age point. Statistical significance is denoted by asterisks, highlighting comparisons between conventional and SPF facilities at each time point(***p < 0.001).
