# Supplementary material for: The Influence of Specific Pathogen-Free and Conventional Environments on the Hematological Parameters of Pigs Bred for Xenotransplantation
Source: Life (Basel). 2024 Sep 8;14(9):1132. doi: 10.3390/life14091132 (PMC11433355; doi:10.3390/life14091132)
Supplement: Supplementary file 1 [file life-14-01132-s001.zip › Table S1.pdf]

Table S1. List of pathogens excluded from SPF pigs.

**Viruses**
African swine fever virus (ASFV)
Alphatorquevirus (Torque teno virus, TTV)
Bovine viral diarrhea virus (BVDV)
Classical Swine Fever (CSFV)
Encephalomyocarditis Virus (EMCV)
Foot and mouth disease virus (FMDV)
Getah virus (GETV)
Hepatitis E Virus (HEV)
Japanese encephalitis virus (JEV)
Porcine astrovirus (PAstV)
Porcine circovirus type 2 (PCV2)
Porcine circovirus type 3 (PCV3)
Porcine cytomegalovirus (PCMV)
Porcine epidemic diarrhea virus (PEDV)
Porcine lymphotrophic herpesvirus (PLHV)
Porcine parvovirus (PPV)
Porcine reproductive and respiratory syndrome virus (PRRSV)
Porcine respiratory coronavirus (PRCV)
Pseudorabies virus (Aujeszky's Disease, ADV)
Rotavirus
Swine Influenza virus (SIV)
Swinepox virus
Transmissible gastroenteritis virus (TGEV)
**Bacteria**
Actinobacillus pleuropneumoniae
Actinobacillus suis
Bordetella bronchiseptica (BB)
Brachyspira pilosicoli
Brachyspira hyodysenteriae
Brucella suis
E.coli (K88)
Erysipelothrix rhusiopathiae
Hemophilus parasuis
Lawsonia intracelluraris
Leptospira spp. (hardjo, pomona, tarrasovi, interogans)
Mycoplasma hyopneumoniae
Mycoplasma hyorhinis
Pasteurella multocida (PM, PmA)
Salmonella
Streptococcus suis
**Parasite**
Eimeria spp.

Isospora suis
Toxoplasma spp.
